# Effects of nitrogen application frequency under integrated water and fertiliser management on soil physicochemical properties, dry matter accumulation, photosynthetic characteristics and yield

**DOI:** 10.1038/s41598-026-50928-x

**Published:** 2026-05-14

**Authors:** Xiaoqian Wu, Jun Zhang, Yuwen Wu, Leru Zhou, Bolang Chen

**Affiliations:** 1 College of Resources and Environment, Xinjiang Agricultural University, Urumqi, 830052 China; 2 Xinjiang Laboratory of Soil and Plant Ecological Processes, Urumqi, 830052 China

**Keywords:** Cotton, Nitrogen application frequency, Dry matter accumulation, Nitrogen accumulation, Yield, Nitrogen recovery efficiency, Ecology, Ecology, Environmental sciences, Plant sciences

## Abstract

Water-fertilizer integration is recognized as a crucial strategy for enhancing crop yield and improving nitrogen recovery efficiency (NRE).However, the specific mechanisms by which the frequency of split nitrogen fertilizer applications influences cotton yield and NRE under water-fertilizer integration conditions remain poorly understood. To determine optimal N fertilization strategies, a two-year consecutive field experiment was conducted from 2023 to 2024, comparing three treatments: no nitrogen fertilizer (CK), nitrogen fertilizer applied in eight follow-up applications (N8) and nitrogen fertilizer applied in ten split applications (N10). The results indicated that the N8 treatment maintained the highest levels of soil organic matter and alkali-hydrolyzable nitrogen content in the 0 ~ 20 cm soil layer compared with other treatments. When nitrogen application rates were consistent, adjusting the frequency of nitrogen application could improve the distribution of dry matter and nitrogen accumulation in cotton within the Xinjiang cotton-growing region. Specifically, the N8 treatment achieved a balanced relationship between vegetative and reproductive growth, facilitating greater accumulation of dry matter and nitrogen in reproductive organs. In both 2023 and 2024, the N8 treatment increased net photosynthetic rate by 25.27% and 45.21%, transpiration rate by 55.30% and 46.42%, and stomatal conductance by 38.89% and 55.95%, seed cotton yield by 31.94% and 36.78%, nitrogen recovery efficiency by 56.07% and 49.11%, respectively, compared to the CK treatment, while intercellular CO₂ concentrations decreased by 18.76% and 22.91%, respectively. Moreover, cotton seed yield is highly significantly and positively correlated with dry matter accumulation and nitrogen accumulation.Under irrigation under plastic film irrigation, applying N fertilizer in eight doses effectively ensured soil nutrient availability, promoted dry matter and nitrogen in cotton, promote photosynthesis, and thereby enhanced cotton yield.

## Introduction

Cotton is a vital cash crop, boasting the highest yield among all fiber crops^1^. Xinjiang is China’s largest commercial cotton-producing region as ample sunshine and optimal accumulated temperature, with its cotton planting area and output accounting for 86.24% and 92.24% of national total, respectively^2^. However, cotton production faces several constraints, particularly related to fertilizer supply. Commercial fertilizers contribute to at least 40% of the productivity achieved in intensive, high-yield cotton cultivation systems^3^. Nevertheless, excessive fertilizer application and unscientific fertilization practices have resulted in nutrient fixation, leaching, and losses, thereby reducing fertilizer utilization efficiency, limiting yield improvements, and hindering the advancement of modern agricultural production^4,5^. Given the scarcity of arable land resources and the demand for high cotton yields, optimizing fertilizer application strategies is one of the most critical approaches to enhancing cotton productively.

Increasing nitrogen is an essential nutrient for crop growth and development^6,7^. Luo et al.^8^ demonstrated that optimizing nitrogen fertilizer supply within the range of 225–300 kg N ha⁻¹ can enhance root foraging capacity, promote nitrogen uptake, increase aboveground biomass, and ultimately improve seed cotton yield. Therefore, the application of nitrogen fertilizer has been emphasized as a critical measure to improve cotton yield. According to crop fertilizer requirements, soil fertilizer supply capacity, and fertilizer performance, adjusting the frequency of nitrogen application can provide a sufficient nitrogen supply throughout the cotton growth cycle, reduce nitrogen losses, improve nitrogen utilization efficiency, maintain soil nitrogen balance, and achieve synergistic improvements in yield and nitrogen fertilizer use efficiency^9,10^. Nitrogen is a key nutrient for plant growth and photosynthesis, and the intensity of photosynthesis directly influences dry matter accumulation and nutrient accumulation in cotton^11^. Thus, proper nitrogen application during the growth process can enhance cotton yield and fiber quality.

Nitrogen is the fundamental element constituting substances such as cotton proteins, chloroplasts, and nucleic acids^12^. In the early growth stages, it promotes root growth, strengthens seedlings, and preserves buds^13^. During the later stages, it strengthens bolls and enhances fiber quality^14^. As the core nutrient in cotton cultivation, nitrogen helps coordinate vegetative and reproductive growth to achieve high yields of premium cotton^15^. There are significant differences in nutrient requirement characteristics of cotton at various growth seasons. So, Rational nitrogen allocation is a key strategy for improving both cotton yield and nitrogen recovery efficiency. For example, Liu et al.^16^ demonstrated that a single application of nitrogen fertilizer decreased the cotton harvest index and seed cotton yield. Guo et al.^17^ reported that splitting nitrogen applications into 40% at pre-planting, 15% at the square grain stage, 23% at the primordial stage, and 22% at the full flower stage resulted in the highest cotton yield, with the lowest abscission rate. Li et al.^18^ also found that 70% fertilization at the flowering stage can promote nitrogen uptake and utilization efficiency of cotton with a nitrogen application rate of 270 kg ha^− 1^. However, Yang et al.^19^ reported that the highest biomass and yield were achieved with a nitrogen application rate of 225 kg ha^− 1^, split as 0% at pre-planting, 40% at first flowering and 60% at full bloom. Similarly, the study of Raphael et al.^20^ has proved that nitrogen fertilization in the later growth stage of cotton will affect the production of assimilates, thus improving seed cotton yield. Therefore, it is important to determine an appropriate nitrogen application frequency in improving cotton yield and reducing nitrogen loss.

Applying fertilizer at the right time and crop growth stage ensures an adequate nutrient supply when crop needs it, while also preventing fertilizer waste and environmental pollution^21^. Drip irrigation fertilization technology is the best method for achieving real-time, precise nutrient delivery^22,23^. In Xinjiang, the mulched drip irrigation technique with integrated water and fertilizer management is the main cultivation method used to boost cotton yield and improve fertilizer use efficiency^24,25^. Applying nitrogen through drip irrigation beneath plastic mulch in Xinjiang has been shown to significantly increase cotton yields by approximately 43.38% compared to unfertilized fields, ensuring consistently high productivity^26,27^. Uzen et al.^28^ found that an appropriate nitrogen application frequency facilitated cotton yield accumulation under a fixed nitrogen rate. However, under drip irrigation conditions, it remains to be thoroughly investigated how to determine both the frequency of nitrogen application and the optimal distribution ratio per application to concurrently enhance cotton yield, improve nitrogen recovery efficiency, and reduce labor costs.

In this study, we examined soil physico-chemical properties, cotton nitrogen uptake and allocation, and yield under varying nitrogen fertilizer application frequencies. The objectives of this study were to (1) clarify the effects of nitrogen fertilizer frequency on the soil environment, (2) uncover the regulatory mechanisms of nitrogen fertilizer frequency on cotton nitrogen uptake and yield, and (3) offer an empirical foundation for improving cotton production through integrated water-fertilizer technology in arid and semi-arid regions.

## Materials and methods

### Experimental field profile

The experiment was conducted under field conditions from 2023 to 2024 at the cotton experimental station (44°10′E, 86°58′N) in Hutubi County, northern Xinjiang. The region experiences a temperate continental arid and semi-arid climate, and cotton cultivation follows a continuous cropping and annual ripening system. The field soil was a gray desert loam, with the following properties in the 0~ 20 cm layer, 11.54 g kg^− 1^ organic matter, 22.4 mg kg^− 1^ alkaline N, 18.4 mg kg^− 1^ Olsen-P, 207.6 mg kg^− 1^ exchangeable potassium, and pH 8.26.

### Experimental design and field management

Based on the previous reports^29,30^ and in combination with the local actual conditions, the methods of applying nitrogen fertilizer in 8 sessions and 10 sessions were selected respectively, three nitrogen fertilizer application frequency treatments were established: no nitrogen fertilizer (CK), nitrogen fertilizer applied in 8 doses (N8) and nitrogen fertilizer applied in 10 times (N10), with each treatment was replicated 3 times, resulting in a total of 9 monitoring plots, each measuring 10 m × 6.9 m = 69 m^2^. Fertilizer and irrigation were applied uniformly to ensure that the total amounts of fertilizer irrigation remained consistent across treatments. The irrigation quota during cotton growing season was 450 mm, with 10 irrigation events in all cases. Nitrogen fertilizer was applied as urea (the nitrogen content is 46%), with 20% used as a basal fertilizer before sowing and the remaining 80% applied via fertilization (see Table 1). The N8 treatment involved one top-dressing application during the seedling stage, three applications during the budding stage, four applications during the blooming stage, and no top-dressing during the boll opening stage. The N10 treatment comprised two top-dressing applications during the seedling stage, three applications during the budding stage, four applications during the blooming stage, and one application during the boll opening stage(see Table 2). Phosphate and potash were supplied as heavy calcium superphosphate (the content of P_2_O_5_ is 46%) and potassium sulfate (the content of K_2_O is 52%), respectively, all applied as basal fertilizers. The total nutrients inputs were 300 kg N ha^−1^, 150 P_2_O_5_ ha^−1^, 90 kg K_2_O ha^−1^. the test crop was Jinken 1441.The sowing and harvest dates for 2023 are April 11th and September 4th respectively, with a growth period of 146 days; for 2024, the sowing and harvest dates are April 22nd and September 25th respectively, with a growth period of 156 days. The planting method was drip irrigation under the plastic film mulch, with a row spacing of (66 + 10) cm, a plant spacing of 9.2 cm, and a planting density of 19.0 × 10^4^ plants ha^−1^. Field management practices were consistent with those used in local cotton production.

**Table 1:** Nitrogen application rate under different treatments.

Treatment	Nitrogen application rate/kg hm^−2^											
	Base fertilizer	Topdressing										
N8	60	0	16.8	24	24	48	48	36	24	19.2	0	
N10	60	16.8	16.8	19.2	19.2	43.2	43.2	28.8	19.2	16.8	16.8	

**Table 2:** Cotton fertilization periods and dates for 2023–2024.

Year	Base fertilizer	Topdressing									
	Before sowing	Seedling stage	Budding stage			Blooming and boll-forming stage					
2023	4.10	6.9	6.16	6.24	7.3	7.9	7.16	7.21	7.28	8.4	8.10
2024	4.22	6.12	6.20	6.28	7.6	7.14	7.20	7.28	8.5	8.11	8.17

### Determination standard and method

#### Soil pH, conductivity, organic matter and alkali-hydrolyzable nitrogen^31^content determination

Soil samples were collected from 0~20 cm and 20~40 cm soil layers of each plot during the bud, blooming and boll opening stages of cotton, the samples were air-dried naturally, thoroughly mixed, and sieved for analysis. Soil pH and electrical conductivity were measured using the electrode method; organic matter content was determined by the external heating method with potassium dichromate; Alkali-hydrolyzable nitrogen content was measured using the alkaline hydrolysis diffusion method.

#### Determination of plant dry matter and nitrogen^31^content

During the bud, blooming, and boll opening stages, 5 representative cotton plants were randomly sampled from each plot, the plants were separated into roots, stems, leaves, and reproductive organs, and were first heated at 105 ℃ for 30 min to halt enzymatic activity and then dried at 80 ℃ until a constant weight was reached. The dried plant tissues were ground using a pulverizer, and total nitrogen content was determined by the semi-micro Kjeldahl method.

#### Photosynthetic parameters and SPAD values

From each experimental plot, three plants in the central two rows were randomly selected, and the fourth-leaf-from-the-base of each cotton plant was measured. The photosynthetic parameters (net photosynthetic rate, intercellular CO₂ concentration, transpiration rate, and stomatal conductance) were measured using the LCi T/LCpro T photosynthesis analyzer (ADC Bio Scientific, UK) on clear, windless mornings with ample natural light between 11:00 AM and 1:00 PM during the budding and blooming stage (July 12, 2023, and July 11, 2024) and the blooming and boll-forming stage (August 13, 2023, and August 9, 2024). The SPAD value was also measured on the fourth-leaf-from-the-base of cotton plants using an SPAD-502 chlorophyll meter. Three leaf sections were sampled from different locations, and the average value was taken to characterize the chlorophyll content of the treated cotton leaves.

#### Yield determination^32^

During the cotton boll opening period, the number of cotton plants and bolls was recorded in a 6.67 m^2^ sampling area within each plot. 30 bolls were randomly harvested from the upper, middle, and lower parts of the cotton plants to determine the average boll weight, cotton yield was calculated based on plant density, boll number per plant, and average boll weight, with a yield coefficient of 90% was applied to account for seed cotton conversion.

1 $${\text{Seed cotton yield}}\left( {{\mathrm{kg}}\cdot{\mathrm{h}}{{\mathrm{m}}^{ - {\mathrm{2}}}}} \right) = {{\mathrm{N}}_{{\mathrm{ph}}}} \times {{\mathrm{N}}_{{\mathrm{bpp}}}} \times {\mathrm{BC}} \times {\mathrm{9}}0\%$$

(where N_ph_ denotes number of plants harvested; N_bpp_ denotes number of bolls per plant; BC denotes boll count).

#### Nitrogen recovery efficiency^33^

2 $${\text{Nitrogen recovery efficiency}}\left( \% \right) = \left( {{\mathrm{N}} - {{\mathrm{N}}_0}} \right){\text{ }}/{{\mathrm{F}}_{\mathrm{t}}}$$

(where N is total N uptake by cotton in fertilized plots, N0 is total N uptake by cotton in unfertilized (control) plots, and F_t_ is the amount of N fertilizer applied).

### Data processing and statistical methods

Microsoft Excel 2016 was used for data processing. For each treatment, three replicates were set up as independent experimental units. Soil samples from the same plot were combined into a single composite sample, and five plants from each plot were combined into a single sample for analysis; all measurement results for yield-related indicators within each plot were aggregated. Significance tests for photosynthetic parameters and SPAD values were conducted using subsamples.

Given that the experiment involved multiple factors—including year, treatment, soil depth, and growth stage—and that there were significant interactions among these factors, all statistical analyses were conducted separately for each year, growth stage, and soil layer. One-way analysis of variance (ANOVA) was performed with SPSS 25.0 software. The significance of differences between treatments was compared with the new complex extreme deviation test (Duncan’s method), and plotted with Origin 2021 software.

## Results

### Effect of frequency of nitrogen application on soil chemical properties

#### Effect of frequency of nitrogen application on soil pH

**Fig. 1:**
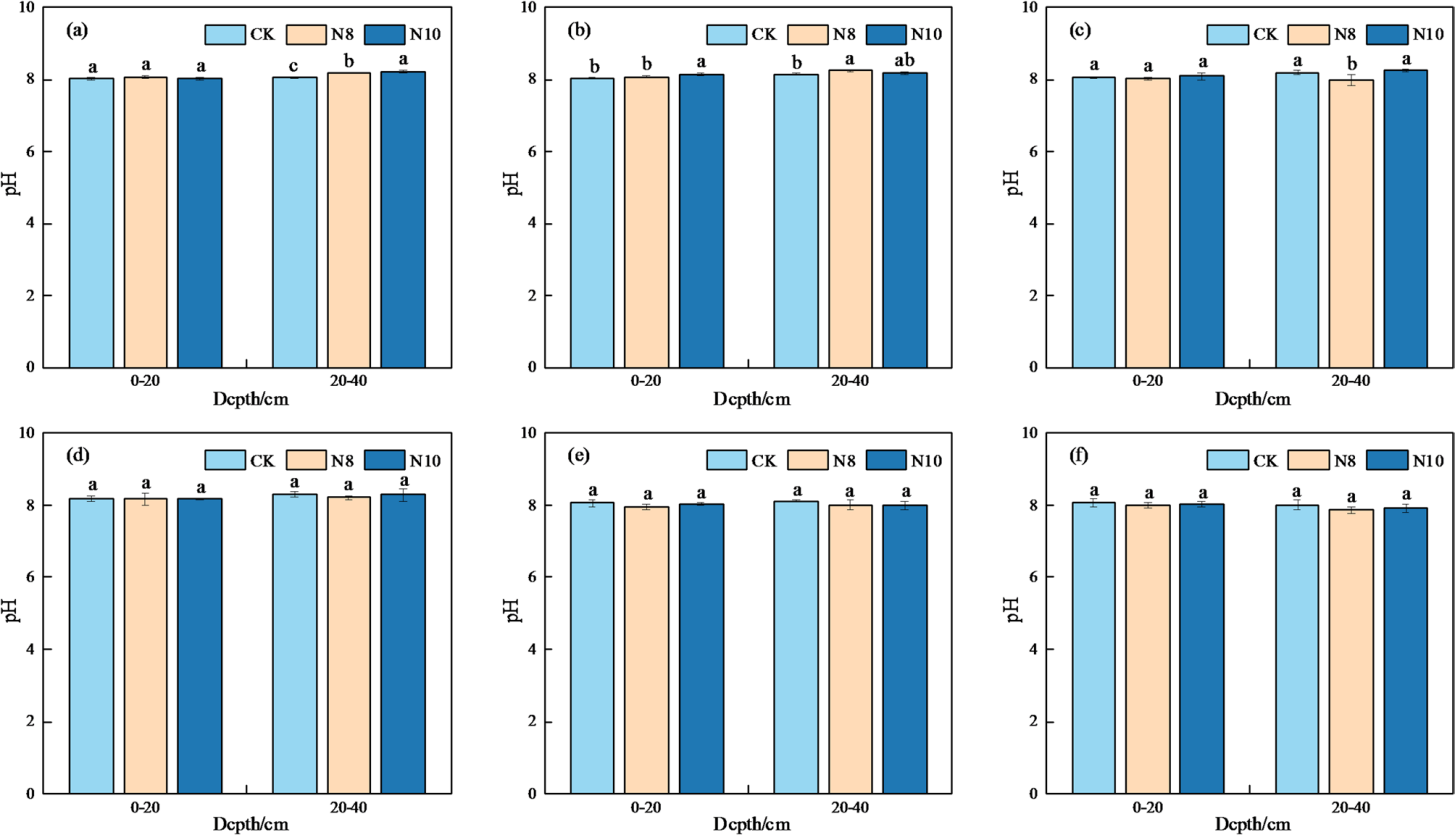
Soil pH changes at different growth stages of cotton under different nitrogen application frequencies from 2023 to 2024. Figure **a**, **b** and **c** represent cotton budding stage, blooming stage and boll opening stage in 2023 respectively; **d**, **e **and **f **stand for cotton budding stage, blooming stage and boll opening stage in 2024, respectively. The different lowercase letters in the same column indicate significant differences for the different nitrogen application frequencies (*p* < 0.05).

The impacts of nitrogen application frequency on soil pH across different soil layers during various fertility stages of cotton are depicted in Fig. 1. At the budding stage: in 2023, no significant differences were observed in the pH levels of the 0~20 cm soil layer among the treatments; the pH of the 20 ~ 40 cm soil layer in the N10 treatment was significantly higher than that of the other treatments, and the N8 treatment showed a significantly higher pH compared to the CK treatment. In 2024, there were no significant variations in the pH values of both the 0~20 cm and 20~40 cm soil layers across all treatments.

At the blooming stage: in 2023, the pH of the 0~20 cm soil layer in the N10 treatment was significantly higher than that in the other treatments, while no significant difference was found between the CK and N8 treatments; for the 20~40 cm soil layer, the N8 treatment had a significantly higher pH than the CK and N10 treatments, whereas no significant difference was observed between the CK and N10 treatments. In 2024, no significant differences were detected in the pH levels of the 0~20 cm and 20~40 cm soil layers among the various treatments.

At the boll opening stage: in 2023, the pH of the 0~20 cm soil layer did not show significant differences among the treatments; however, the pH of the 20~40 cm soil layer in the N8 treatment was significantly lower than that in the other treatments, with no significant difference observed between the CK and N10 treatments. In 2024, no significant differences were found in the pH levels of the 0~20 cm and 20~40 cm soil layers across all treatments.

#### Effect of frequency of nitrogen application on soil conductivity

**Fig. 2:**
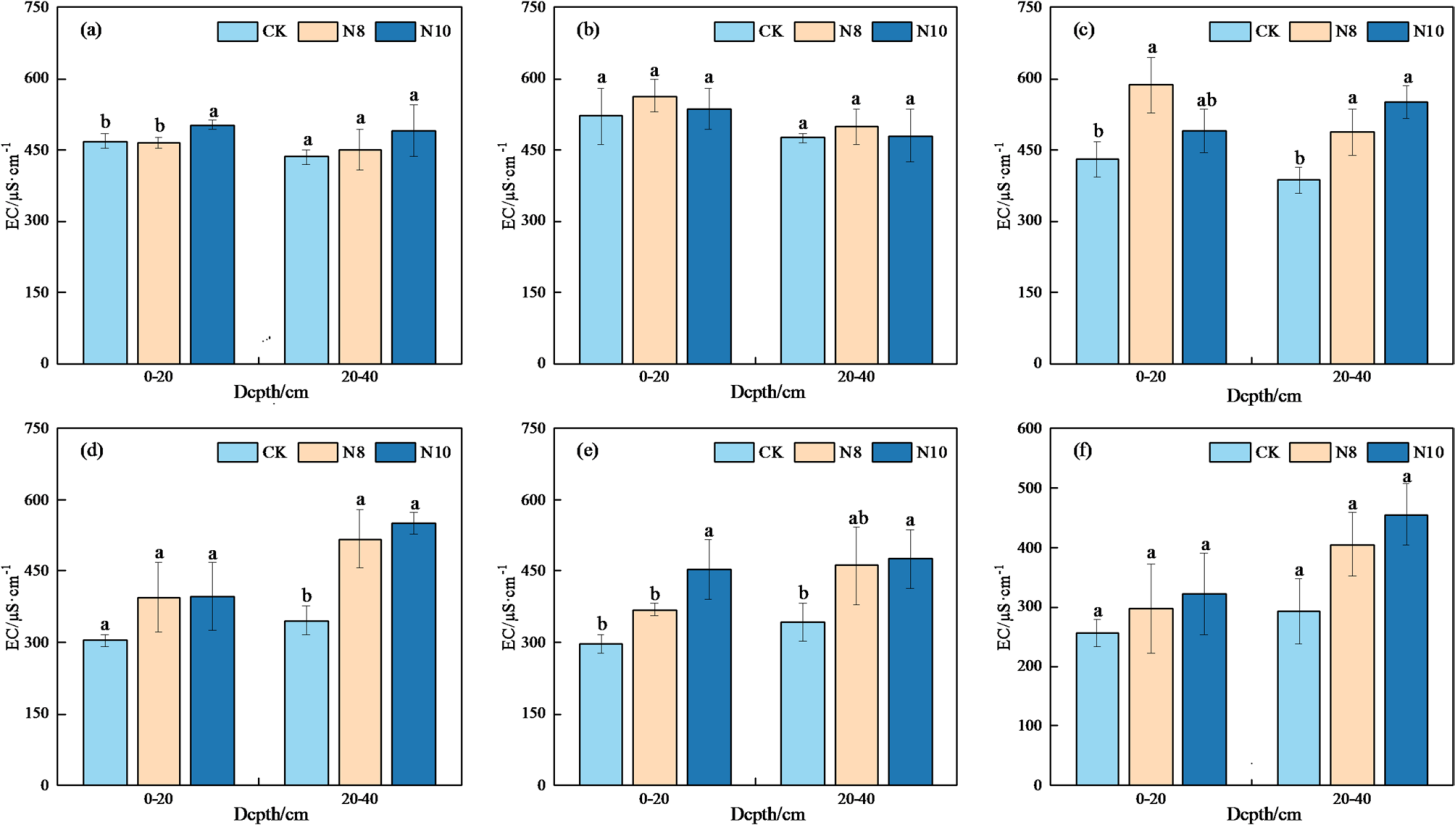
Changes of soil conductivity at different growth stages of cotton under different nitrogen application frequencies from 2023 to 2024.Figure **a**, **b** and **c** represent cotton budding stage, blooming stage and boll opening stage in 2023 respectively; **d**, **e** and **f **stand for cotton budding stage, blooming stage and boll opening stage in 2024, respectively.The different lowercase letters in the same column indicate significant differences for the different nitrogen application frequencies (*p* < 0.05).

The effects of nitrogen application frequency on soil conductivity in different soil layers during cotton fertility stages (Fig. 2). At the budding stage: in 2023, in the 0 ~ 20 cm soil layer, conductivity was significantly higher under the N10 compared to other treatments, and no significant difference was observed between CK and N8; in the 20 ~ 40 cm soil layer, there was no significant difference in conductivity was detected among treatments. In 2024, the N8 and N10 treatments increased electrical conductivity in the 0 ~ 20 cm and 20 ~ 40 cm soil layers compared to CK.

At the blooming stage: in 2023, there was no significant difference in conductivity in either the 0 ~ 20 cm or 20 ~ 40 cm soil layers among treatments. In 2024, in the 0 ~ 20 cm and 20 ~ 40 cm soil layers, conductivity was significantly higher under N10 compared to CK.

At the boll opening stage: in 2023, in both the 0 ~ 20 cm and 20 ~ 40 cm soil layers, conductivity was significantly higher under N8 compared to CK, with no significant difference between N8 and N10. In 2024, there was no significant difference in conductivity in either the 0 ~ 20 cm or 20 ~ 40 cm soil layers among treatments.

#### Effect of frequency of nitrogen application on soil organic matter content

**Fig. 3:**
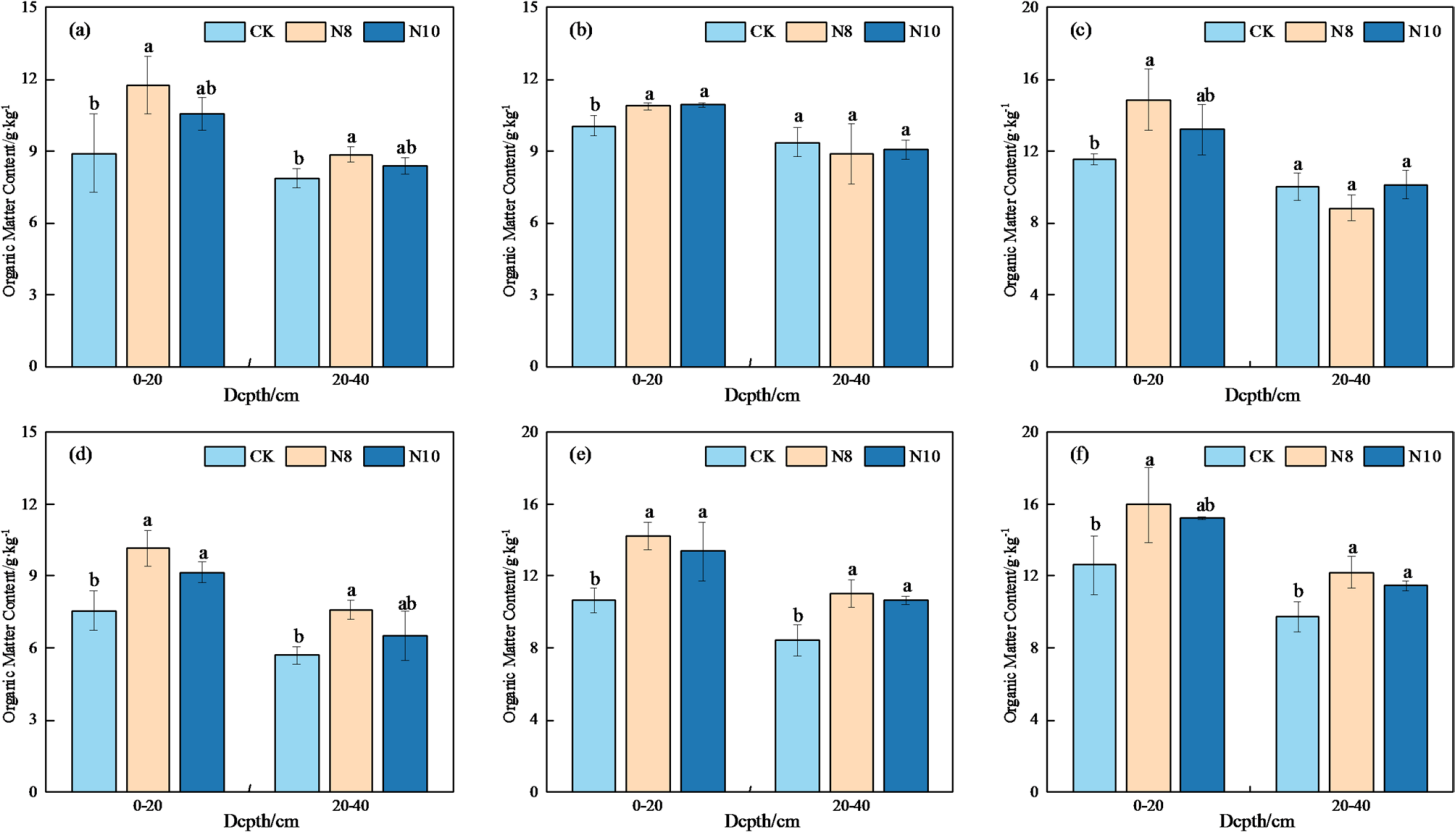
Changes of soil organic matter at different growth stages of cotton under different nitrogen application frequencies from 2023 to 2024.Figure **a**,** b** and **c** represent cotton budding stage, blooming stage and boll opening stage in 2023 respectively; **d**,** e** and** f** stand for cotton budding stage, blooming stage and boll opening stage in 2024, respectively.The different lowercase letters in the same column indicate significant differences for the different nitrogen application frequencies (*p* < 0.05).

The effect of nitrogen application frequency on soil organic matter in different soil layers during various fertility periods of cotton is depicted in Fig. 3.

In 2023, the organic matter content in the 0–20 cm soil layer under the N8 treatment was 31.90%, 8.10%, and 28.30% higher than that under CK at the budding, blooming and boll opening stages, respectively; only at the budding stage did it significantly increase the organic matter content in the 20–40 cm soil layer by 12.32%. In 2024, compared with CK, the organic matter content in the 0–20 cm soil layer under the N8 treatment increased by 34.15%, 33.97%, and 26.50% at the budding, blooming, and boll opening stages, respectively; similarly, the organic matter content in the 20–40 cm soil layer increased by 32.92%, 31.36%, and 25.34% at the same three stages, respectively.

#### Effect of frequency of nitrogen application on soil alkali-hydrolyzable nitrogen content

**Fig. 4:**
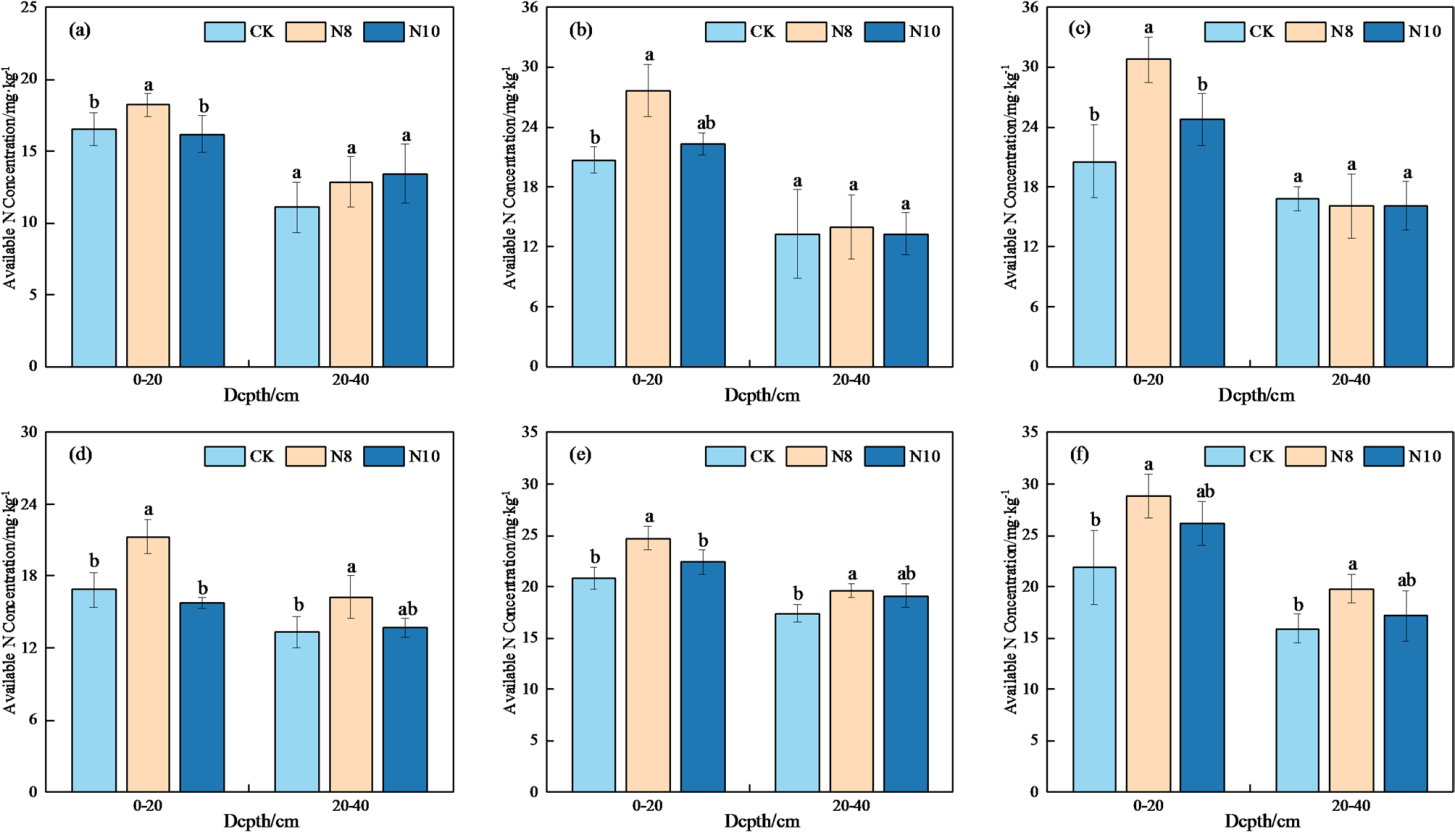
Changes of soil alkali-hydrolyzed nitrogen in different growth periods of cotton under different nitrogen application frequencies from 2023 to 2024.Figure **a**, **b** and **c** represent cotton budding stage, blooming stage and boll opening stage in 2023 respectively; **d**, **e** and** f **stand for cotton budding stage, blooming stage and boll opening stage in 2024, respectively.The different lowercase letters in the same column indicate significant differences for the different nitrogen application frequencies (*p* < 0.05).

The effects of nitrogen application frequency on soil alkali-hydrolyzable nitrogen (AN) in different soil layers during cotton fertility stages (Fig. 4).

In 2023, compared to CK, the alkali-hydrolyzable nitrogen in the 0 ~ 20 cm soil layer under the N8 treatment was significantly increased by 10.11%, 33.50% and 49.61% at the budding, blooming and boll opening stages, respectively; there were no significant differences in alkali-hydrolyzable nitrogen content among treatments in the 20 ~ 40 cm soil layer.In 2024, compared to CK, the alkali-hydrolyzable nitrogen in the 0 ~ 20 cm soil layer under the N8 treatment was significantly increased by 26.35%, 19.10% and 31.62% at the budding, blooming and boll opening stages, respectively, and in the 20 ~ 40 cm soil layer, it was significantly increased by 21.78%, 12.75% and 24.18% at the same three stages, respectively.

### Effect of frequency of nitrogen application on dry matter and nitrogen accumulation in cotton

#### Effect of frequency of nitrogen application on dry matter accumulation

**Fig. 5:**
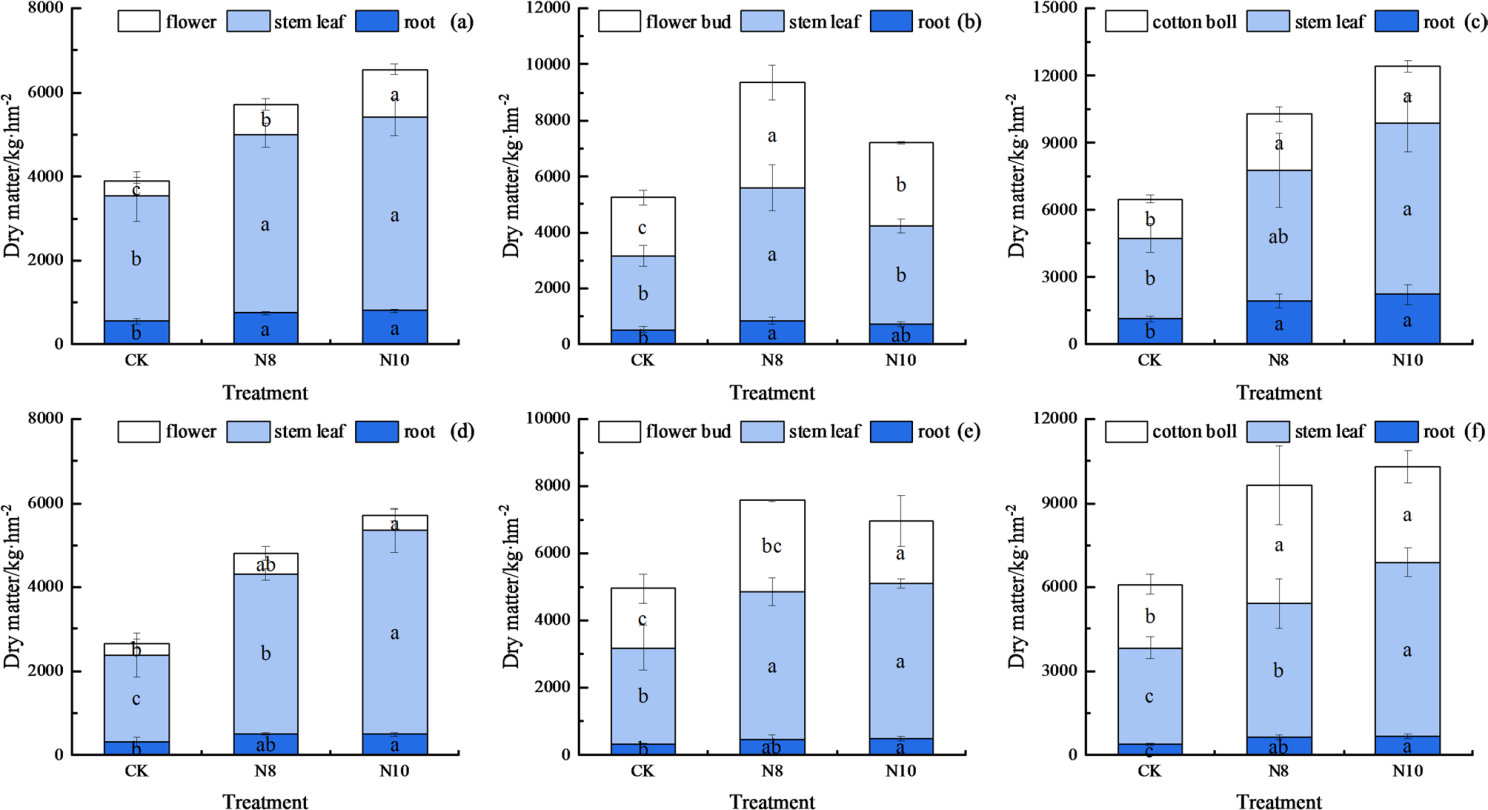
Changes of dry matter accumulation in cotton at different growth stages under different nitrogen application frequencies from 2023 to 2024.Figure **a, b** and** c** represent cotton budding stage, blooming stage and boll opening stage in 2023 respectively; **d**, **e** and **f** stand for cotton budding stage, blooming stage and boll opening stage in 2024, respectively. The different lowercase letters in the same column indicate significant differences for the different nitrogen application frequencies (*p* < 0.05).

As shown in Fig. 5, the total dry matter accumulation in cotton organs across different growth stages was recorded in 2023 and 2024. In 2023, the dry matter accumulation ranged from 3902.4 to 6553.1 kg·hm^−2^at budding stage, 5252.4 to 9370.1 kg·hm^− 2^ at the blooming stage, and 6503.28 to 12415.4 kg·hm^−2^at the boll opening stage. In 2024, these values were 2654.3 to 5699.6 kg·hm^− 2^, 4960.3 to 7573.6 kg·hm^− 2^ and 6086.6 to 10287.2 kg·hm^− 2^ at bud, blooming, and boll opening stages, respectively. When comparing the N8 treatment to the CK treatment in 2023, dry matter mass of cotton increased by an average of 46.43% at the budding stage, 78.39% at the blooming stage, and 58.14% at the boll opening stage.In 2024, under the N8 treatment, the average increases compared to CK were 80.45% at the budding stage, 52.68% at the blooming stage, and 58.37% at the boll opening stage. Similarly, for the N10 treatment in 2023, the average increase in dry matter compared to CK was 67.92% at budding stage, 37.14% at the blooming stage and 90.91% at the boll opening stage. In 2024, the N10 treatment resulted in average increases of 114.73% at the budding stage, 40.58% at the blooming stage, and 69.01% at the boll opening stage compared to CK.

#### Effect of frequency of nitrogen application on nitrogen uptake

**Fig. 6:**
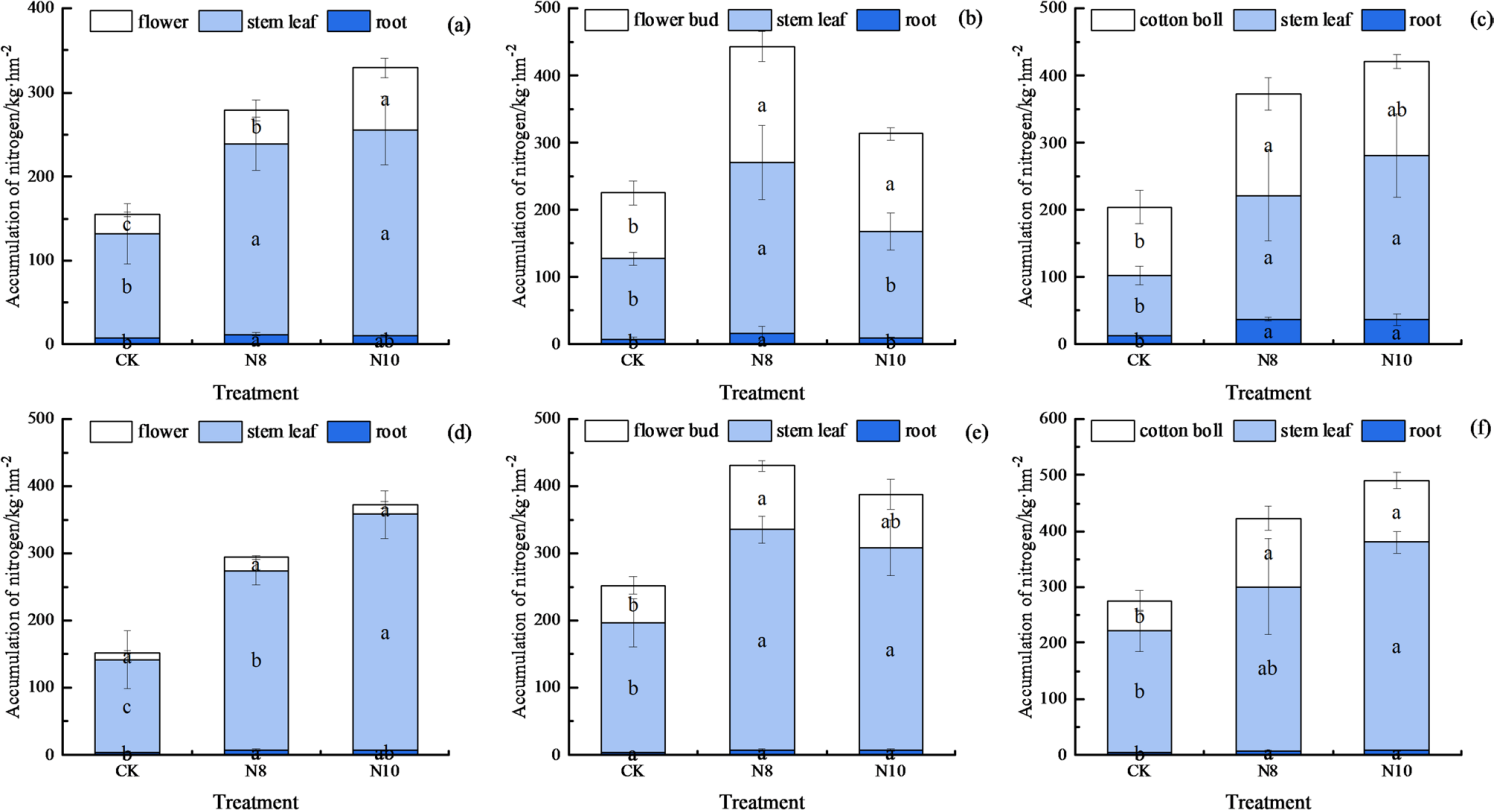
Changes of nitrogen accumulation in cotton at different growth stages under different nitrogen application frequencies from 2023 to 2024.Figure** a**, **b** and** c** represent cotton budding stage, blooming stage and boll opening stage in 2023 respectively; **d,** **e** and **f** stand for cotton budding stage, blooming stage and boll opening stage in 2024, respectively. The different lowercase letters in the same column indicate significant differences for the different nitrogen application frequencies (*p* < 0.05).

As shown in Fig. 6, the total nitrogen accumulation in cotton organs at the bud, blooming, and boll opening stages ranged from 154.84 ~ 331.50 kg·hm^− 2^, 225.65 ~ 443.51 kg·hm^− 2^ and 204.64 ~ 420.58 kg·hm^− 2^ in 2023, respectively; and 152.74 ~ 373.04 kg·hm^− 2^, 252.57 ~ 430.61 kg·hm^− 2^ and 275.84 ~ 489.70 kg·hm^− 2^ in 2024, respectively; under the N8 treatment compared to CK, nitrogen accumulation increased by an average of 80.05% (bud), 96.54% (blooming) and 82.19% (boll opening) in 2023, and 92.66%, 70.48% and 53.40% in 2024, respectively; under the N10 treatment compared to CK, increases averaged 114.08% (bud), 38.86% (blooming) and 105.52% (boll opening) in 2023, and 144.22%, 53.45% and 77.53% in 2024, respectively.

### Effects of frequency of nitrogen application on photosynthetic characteristics and spad values of cotton

#### Effects of frequency of nitrogen application on photosynthetic characteristics

**Fig. 7:**
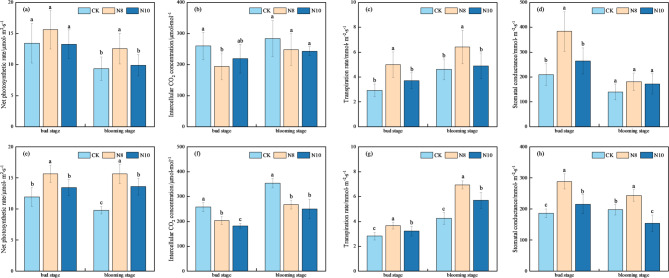
Changes in cotton photosynthetic characteristics during the budding and blooming stage and the blooming and boll-forming stage under different nitrogen application frequencies from 2023 to 2024. Figures **a**, **b**, **c**, and **d **represent net photosynthetic rate, intercellular CO_2_ concentration, transpiration rate, and stomatal conductance of cotton in 2023, respectively; Figures **e**, **f,** **g**, and **h** represent net photosynthetic rate, intercellular CO_2_ concentration, transpiration rate, and stomatal conductance of cotton in 2024, respectively.The different lowercase letters in the same column indicate significant differences for the different nitrogen application frequencies (*p* < 0.05).

The frequency of nitrogen application significantly influenced the photosynthetic characteristics of cotton during the budding and blooming stage and the blooming and boll-forming stage (Fig. 7). In 2023, the net photosynthetic rate, transpiration rate, and stomatal conductance of the N8 treatment were higher than those of other treatments, while no significant differences were observed between the CK and N10 treatments. The intercellular CO₂ concentration in the CK treatment was higher than in other treatments, with no significant difference between the N8 and N10 treatments. In 2024, the net photosynthetic rate, transpiration rate, and stomatal conductance of the N8 treatment were significantly higher than those of other treatments; but the intercellular CO₂ concentration in the CK treatment was significantly higher than in other treatments.

#### Effects of frequency of nitrogen application on SPAD values

**Fig. 8:**
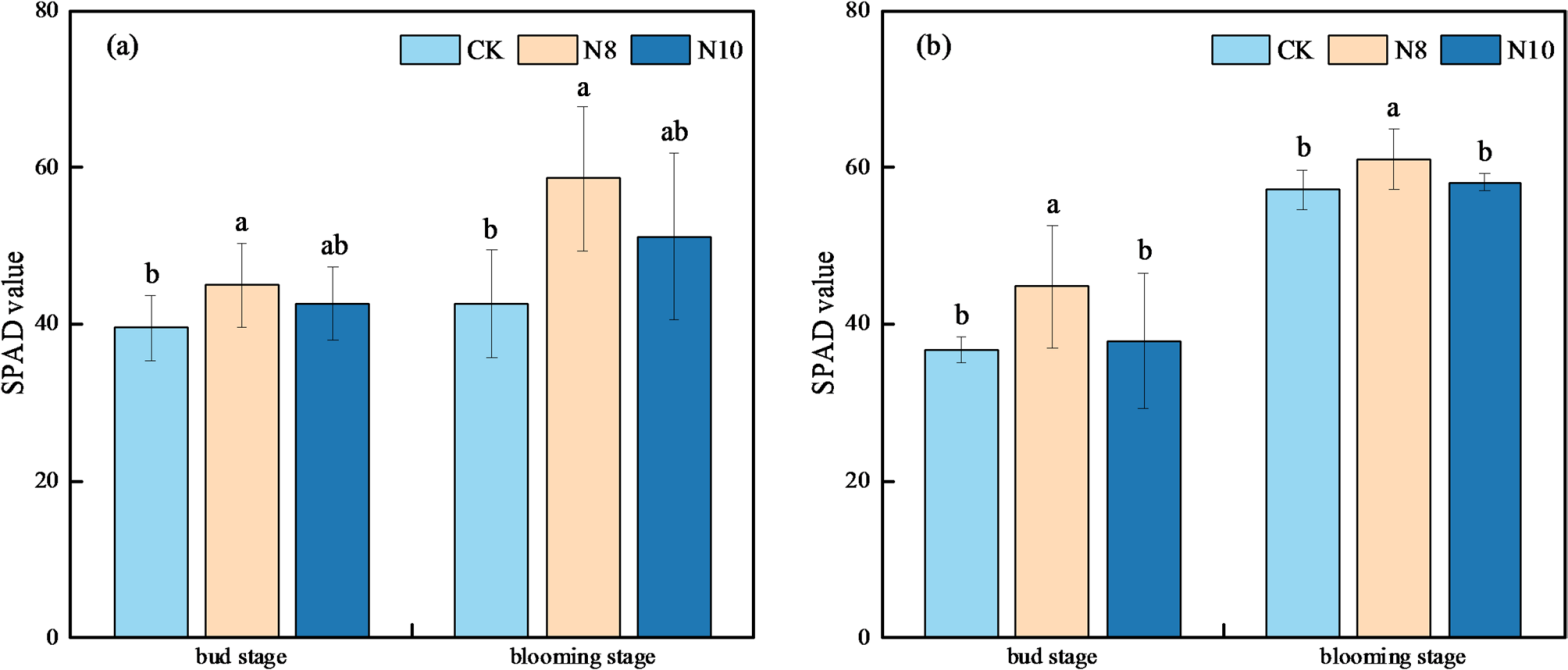
Changes in SPAD values of cotton during the budding and blooming stage and the blooming and boll-forming stage under different nitrogen application frequencies from 2023 to 2024. Figures **a **and** b** represent 2023 and 2024, respectively. The different lowercase letters in the same column indicate significant differences for the different nitrogen application frequencies (*p* < 0.05).

The frequency of nitrogen application significantly influenced SPAD values during the budding and blooming stage and the blooming and boll-forming stage of cotton (Fig. 8). In 2023, SPAD values for the N8 treatment showed no significant difference compared to the N10 treatment, while were significantly higher than the CK treatment, representing increases of 13.71% and 37.21%, respectively. In 2024, the SPAD values of the N8 treatment during the budding and blooming stage and the blooming and boll-forming stage were significantly higher than those of other treatments, compared with the CK treatment, increased by 21.73% and 6.90%, respectively.

### Effect of frequency of nitrogen application on cotton yield, yield components and nitrogen recovery efficiency

In both 2023 and 2024, no significant differences in cotton harvest density were observed among treatments (Table 3). In 2023, the number of cotton bolls per plant under N8 and N10 was significantly higher than that under the CK treatment, and with no significant difference between N8 and N10 treatments. In 2024, the N8 treatment exhibited a significantly higher number of bolls per plant compared to other treatments, while no significant differences were found between the CK and N10 treatments. Regarding cotton boll weight, no significant differences among treatments were observed in 2023, however, in 2024, the N8 treatment showed significantly higher boll weights than other treatments, with no significant difference between CK and N10. In 2023, in terms of seed cotton yield, the N8 treatment significantly outperformed CK, and there was no significant difference between N8 and N10. In 2024, the N8 treatment yielded significantly more than CK. Compared to CK, the N8 treatment increased seed cotton yield by 31.94% in 2023 and 36.78% in 2024. There was no significant difference in nitrogen fertilizer use efficiency between the N8 and N10 treatments in either year.

**Table 3:** Effects of nitrogen application times on cotton yield traits.Data are mean ± standard deviation. Different lowercase letters in the same column indicated significant difference between different treatments in the same year (*P* < 0.05).

Year	Treatment	Number of plants harvested /×10^4^·hm^−2^	Number of bells per plant	Boll count/g	Seed cotton yield/kg·hm^−2^	Nitrogen recovery efficiency/%
2023	CK	17.93 ± 0.76a	4.51 ± 0.20b	4.38 ± 0.30a	3183 ± 274b	
	N8	19.34 ± 1.01a	4.94 ± 0.23a	4.90 ± 0.44a	4200 ± 299a	56.07 ± 17.37a
	N10	18.03 ± 0.92a	5.07 ± 0.11a	4.66 ± 0.37a	3831 ± 360a	71.98 ± 11.87a
2024	CK	15.99 ± 0.83a	4.98 ± 0.47b	4.49 ± 0.09b	3205 ± 165c	
	N8	17.29 ± 0.57a	5.68 ± 0.13a	4.92 ± 0.29a	4384 ± 242a	49.11 ± 22.34a
	N10	17.19 ± 1.36a	5.22 ± 0.18ab	4.67 ± 0.08ab	3770 ± 234b	71.29 ± 7.74a

### Correlation of cotton seed cotton yield with various indicators

**Fig. 9:**
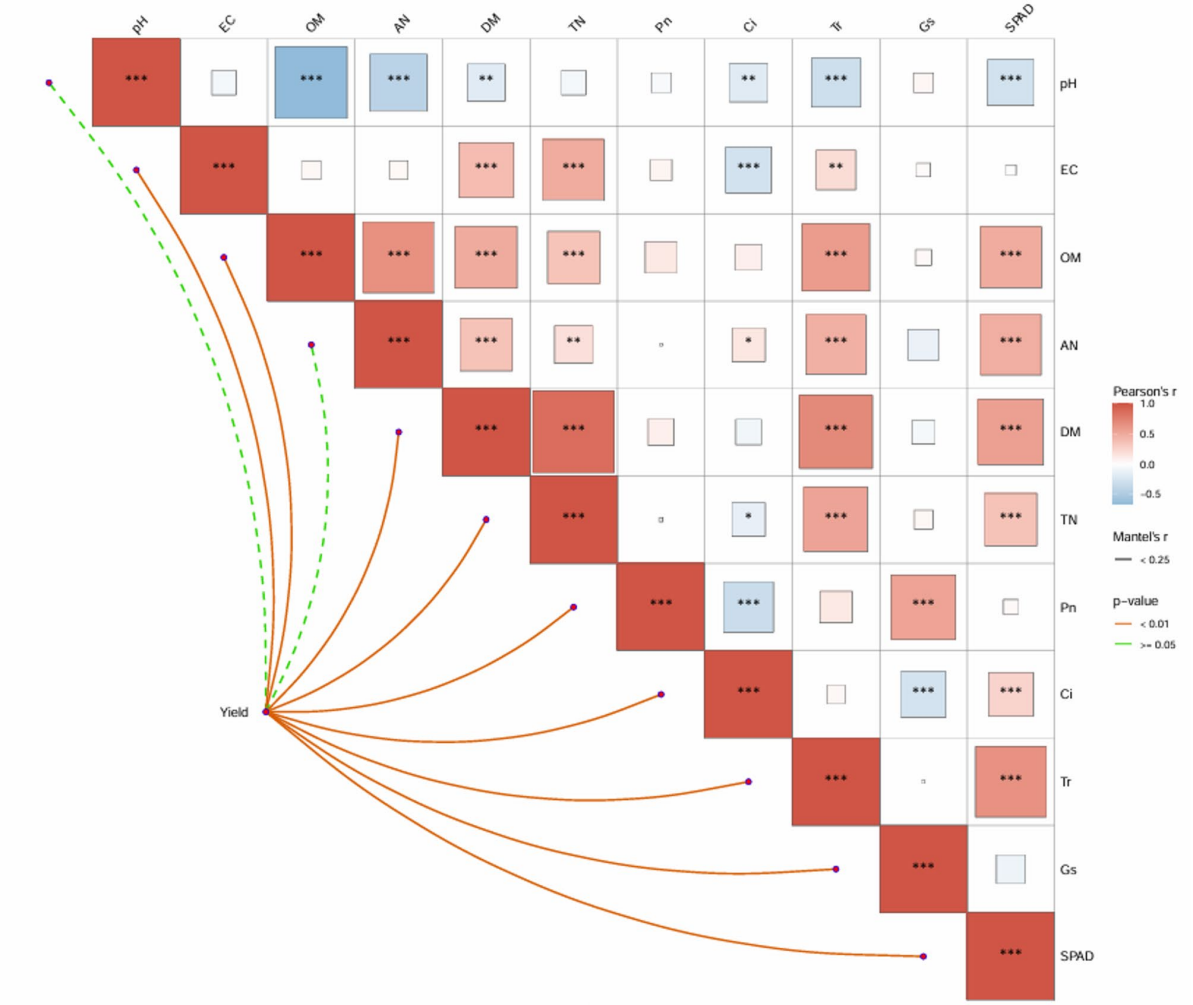
Correlation heat map of between seed cotton yield and each index.The square size indicates the correlation value, and red and blue colors indicate the positive and negative correlations, respectively. *, * *, and * ** represent the P values less than 0.05, 0.01,0.001, respectively.Yield, Seed cotton yield; pH, soil pH; EC, soil conductivity; OM, soil organic matter; AN, soil alkali-hydrolyzed nitrogen; DM, cotton dry matter accumulation; TN, cotton nitrogen accumulation; Pn, net photosynthetic rate; Ci, intercellular CO_2_ concentration; Tr, transpiration rate; Gs, stomatal conductance; SPAD, cotton SPAD value.

The correlation analysis results between cotton yield, soil physicochemical properties, cotton growth indicators, and cotton photosynthetic characteristics are shown in Fig. 9. Statistical analysis indicates that cotton yield shows no statistically significant correlation with pH or alkali-hydrolyzable nitrogen. Conversely, cotton yield exhibited a significant correlation with organic matter content; it also showed significant correlations with dry matter accumulation and nitrogen accumulation. Furthermore, cotton yield demonstrated significant correlations with net photosynthetic rate, intercellular carbon dioxide concentration, transpiration rate, stomatal conductance, and SPAD values.

## Discussion

### Effect of frequency of nitrogen application on soil chemical properties

The nutrient content of soil significantly influences its fertility status, directly affecting cotton yield and quality^34,35^. In this experiment, we observed that the varying N application frequencies had a relatively minor impact on soil pH. This may be attributed to the fact that urea, a water-soluble fertilizer, is typically applied in multiple split doses as a follow-up fertilizer. It is rapidly absorbed by plants and becomes diluted with water, diminishing its inherent acid-base properties, and thus having a limited impact on soil pH. Cai et al.^36^ demonstrated that, compared to a single follow-up fertilizer application, three to four follow-up applications led to a greater concentration of soil NO_3_^−^N in the upper soil layer (0 ~ 40 cm) during the flowering and sizing stages of wheat. Qiu et al.^37^ showed that in the cotton-growing areas of Xinjiang, with an N application rate of 300 kg·hm^− 2^, a fertilization schedule of nine times (20% basal application, 20% in three applications at the budding stage, 30% in three applications at anthesis, and 30% in three applications at full bloom) significantly increased the levels of soil nitrate, ammonium, and alkali-hydrolyzable nitrogen (AN) compared to a no-N fertilization treatment. Zhang et al.^38^ found that in the cotton-growing areas of Xinjiang, excessive water inflow and prolonged high-frequency irrigation were primary factors contributing to carbon leaching under certain conditions. In contrast, this study revealed that under drip irrigation beneath a membrane, the surface soil organic matter and alkali-hydrolyzable nitrogen in the N8 treatment were significantly higher than those in the control (CK) treatment. This environment was conducive to the crop’s absorption of soil nutrients, thus promoting the growth and development of cotton and enhancing yield formation.

### Effect of nitrogen application frequency on dry matter and nitrogen accumulation in cotton

Appropriate dry matter accumulation and its coordinated dynamics are crucial for establishing an optimal population structure and achieving high cotton yields^39–41^. Lu et al.^42^ demonstrated that in the cotton-growing regions of the Yangtze River Basin, regulating fertilizer application timing and nitrogen dosage could maintain balanced nitrogen uptake and utilization by reproductive organs, thereby increasing biomass and ultimately enhancing yields. Ren et al.^29^ reported that, in the cotton-growing areas of the Yellow River Basin, during later growth stages, dry matter accumulation increased by 54.6% and 73.3% in Ningjin and Changyi experimental areas, respectively, under a nitrogen allocation ratio of 3:5:2 compared to a 0:10:0 treatment. Abdelraouf et al.^43^ found that increasing fertilization frequency and shortening intervals in 2014/2015 and 2015/2016 improved nitrogen uptake efficiency, cumulative N uptake and grain N content in both observed and simulated values. This study showed that both N8 and N10 treatments enhanced dry matter accumulation in cotton roots, stems and leaves and reproductive organs. At the budding stage, the N10 treatment exhibited higher total dry matter than N8, during the blooming stage, the N8 treatment increased overall plant dry matter by promoting accumulation in reproductive organs; and at the boll open stage, the N10 treatment boosted total plant dry matter by enhancing accumulation in vegetative organs. These differences may stem from varying nutrient demands across cotton growth stages^44^, and the flowering stage is a critical phase for reproductive growth, the N8 treatment supplied high nitrogen during this period to meet nutrient demand, ensuring sufficient nitrogen availability, may accelerate the transition to reproductive growth, and facilitating nitrogen uptake and redistribution from vegetative to productive organs. The N10 treatment applied nitrogen later in the flowering stage, sustaining vigorous vegetative growth and may reduce nitrogen transfer efficiency to seeds, which hindered nutrient translocation from leaves and stems^45^. Therefore, appropriate nitrogen application rates and allocation ratio are essential for optimizing plant dry matter accumulation.

Nitrogen uptake and utilization are prerequisites for dry matter accumulation, which forms the basis for high cotton yields^46–48^.Uzen et al.^28^ showed that moderate fertilization frequency significantly enhanced total nitrogen uptake compared to high or low frequencies. Feng et al.^49^ reported that in the cotton-growing areas of the Yellow River Basin, apparent nitrogen loss was 48.3% under single nitrogen application but reduced it to 38.5% with split applications. This study showed that both N8 and N10 treatments increased nitrogen accumulation in cotton roots, stems, leaves, and reproductive organs, the N8 treatment may effectively balance vegetative and reproductive growth, promoting nitrogen accumulation in reproductive organs and facilitating photosynthate translocation. Wang et al.^50^ noted that while nitrogen content increased with fertilizer dosage, excessive nitrogen slowed or reduced nitrogen accumulation, highlighting the importance of optimal nitrogen application for maximizing accumulation rates. The present study showed that the N10 treatment at the budding stage exhibited higher N accumulation than N8; the N8 treatment at the blooming stage enhanced total plant nitrogen accumulation by increasing uptake in both vegetative and reproductive organs; the N10 treatment at the boll open stage increased the N accumulation of the whole cotton plant by promoting uptake in vegetative organs.

### Effect of nitrogen application frequency on photosynthetic characteristics and SPAD values in cotton

Cotton yield exhibits a strong correlation with plant growth and development indicators, and leaf photosynthetic properties are significantly correlated with plant growth, development, and dry matter accumulation. Tian et al.^10^ demonstrated that in the cotton-growing areas of Xinjiang, during the late growth stage of cotton, adjusting nitrogen application frequency and proportion can increase net photosynthetic rate, transpiration rate, and stomatal conductance while reducing intercellular CO₂ concentration.The results of this study indicate that under the N8 treatment, cotton exhibited higher net photosynthetic rates, transpiration rates, and stomatal conductance compared to the CK and N10 treatments, while intercellular CO₂ concentrations were lower than those in the CK treatment. This suggests that varying nitrogen application frequencies exert distinct effects on cotton photosynthesis, with the application of nitrogen fertilizer in eight installments more effectively promoting photosynthetic activity. Nitrogen fertilization increased net photosynthetic rate, transpiration rate, and stomatal conductance while decreasing intercellular CO₂ concentration, consistent with studies by Dai et al.^51^ and Li et al.^52^ in the cotton-growing areas of the Yellow River Basin. This may occur because under nutrient-sufficient conditions, stomata open to enhance crop respiration, allowing CO₂ to enter plant cells. This may reduce intercellular CO₂ concentration, thereby increasing photosynthetic rate, and ultimately boosting cotton photosynthetic capacity. Under the experimental conditions, the SPAD values of the N8 treatment were significantly higher than those of the CK treatment, while the N10 treatment showed no significant difference from the CK treatment. This indicates that an appropriate nitrogen application frequency may promote the vegetative growth of cotton, increase chlorophyll content in cotton leaves, and lay a material foundation for high cotton yields^53^.

### Effect of different frequency of nitrogen application on cotton yield, yield components and nitrogen recovery efficiency

Nitrogen nutrition plays a critical role in regulating the cotton yield formation, improper fertilizers leads to excessive vegetative growth or nutrient deficiency, which are associated with reduced boll number, boll weight and yield.Conversely, rational nitrogen management coordinates vegetative and reproductive growth, ensuring high-yield and high-quality cotton production^54–56^.Yang et al.^57^ found that in the Yangtze River Basin cotton region, one-time fertilization yielded similar results to traditional three-time fertilization but outperformed two-time fertilization. Luo et al.^58^ in the cotton region of the Yangtze River Basin, showed that among all one-time fertilization schedules, the one-time application of fertilizer to the first flower stage maximized yield, comparable to traditional three-time applications. In this study, N8 and N10 treatments increased seed cotton yield by raising boll number per plant and boll weight, with N8 being the most effective compared to the CK treatment under a total pure N rate of 300 kg·hm^− 2^.

Nitrogen recovery efficiency is a key metric reflecting crop nitrogen uptake and utilization^59,60^. Cai et al.^36^ showed that splitting follow-up nitrogen applications 3–4 times, with about 16.7% applied at the flowering and the irrigating stages, significantly improved yield, WUE (Water Use Efficiency), PFPN (Partial Productivity of Fertilizer with Nitrogen), and NHI (Nitrogen Harvest Index). In this study, the N10 treatments slightly improved N fertilizer utilization efficiency compared to N8, though the difference was not statistically significant.

## Conclusions

In summary, nitrogen frequency significantly influenced soil nutrient content, dry matter and nitrogen accumulation, photosynthetic characteristics, yield traits and nitrogen recovery efficiency in Xinjiang cotton fields. The N8 treatment maintained high soil organic matter and alkali-hydrolyzable nitrogen levels throughout the reproductive period. Adjusting nitrogen application frequency under consistent nitrogen rates optimized dry matter and nitrogen distribution, promote photosynthesis, enhancing then efficiency and yield. Under the experimental conditions, when the total nitrogen application rate was 300 kg·ha^−1^, drip fertilization with water in 8 split applications (N8) yielded the best results.

## Data Availability

The original contributions presented in this study are included in the article. Further inquiries can be directed to the corresponding author.

## Declarations

### Disclaimer

The statements, opinions and data contained in all publications are solely those ofthe individual author(s) and contributor(s) and not of MDPI and/or the editor(s). MDPI and/or the editor(s) disclaimresponsibility for any injury to people or property resulting from any ideas, methods, instructions or products referredto in the content.
